# The effect of prophylactic bilateral salpingectomy on ovarian reserve in patients who underwent laparoscopic hysterectomy

**DOI:** 10.1186/s13048-021-00825-w

**Published:** 2021-06-29

**Authors:** Shizhuo Wang, Jiahui Gu

**Affiliations:** Department of Obstetrics and Gynecology, Shengjing Hospital of China Medical University, 36 San Hao Street, Heping District, Liaoning 110004 Shenyang, China

**Keywords:** Prophylactic bilateral salpingectomy, Laparoscopic hysterectomy, Ovarian reserve

## Abstract

**Background:**

Bilateral salpingectomy has been proposed to reduce the risk of ovarian cancer, but it is not clear whether the surgery affects ovarian reserve. This study compares the impact of laparoscopic hysterectomy for benign disease with or without prophylactic bilateral salpingectomy on ovarian reserve.

**Methods:**

Records were reviewed for 373 premenopausal women who underwent laparoscopic hysterectomy with ovarian reserve for benign uterine diseases. The serum anti-Müllerian hormone (AMH), follicle-stimulating hormone (FSH), luteinizing hormone (LH), estradiol (E2), and three-dimensional antral follicle count (AFC) were assessed before surgery and 3 and 9 months postoperatively to evaluate ovarian reserve. Patients were divided into two groups according to whether they underwent prophylactic bilateral salpingectomy. The incidence of pelvic diseases was monitored until the ninth month after surgery.

**Results:**

There was no significant difference between the two surgery groups in terms of baseline AMH, E2, FSH, LH, and AFC (all *P* > 0.05). There was no difference in potential bias factors, including patient age, operative time, and blood loss (all *P* > 0.05). There was also no significant difference between the two groups 3 months after surgery with respect to AMH (*P* = 0.763), E2 (*P* = 0.264), FSH (*P* = 0.478), LH (*P* = 0.07), and AFC (*P* = 0.061). Similarly, there were no differences between groups 9 months after surgery for AMH (*P* = 0.939), E2 (*P* = 0.137), FSH (*P* = 0.276), LH (*P* = 0.07) and AFC (*P* = 0.066). At 9 months after the operation, no patients had malignant ovarian tumors. The incidences of benign ovarian tumors in the salpingectomy group were 0 and 2.68 % at 3 and 9 months after surgery, respectively, and the corresponding values in the control group were 0 and 5.36 %. The incidences of pelvic inflammatory disease in the salpingectomy group were 10.72 and 8.04 % at 3 and 9 months after surgery, respectively, while corresponding values in the control group were 24.13 and 16.09 %.

**Conclusions:**

Prophylactic bilateral salpingectomy did not damage the ovarian reserve of reproductive-age women who underwent laparoscopic hysterectomy. Prophylactic bilateral salpingectomy might be a good method to prevent the development of ovarian cancer. Larger clinical trials with longer follow-up times are needed to further evaluate the risks and benefits.

## Background

Ovarian cancer is the fifth most common lethal cancer in women. It usually shows a poor prognosis, with diagnosis at a late stage due to its occult symptoms and rapid progression [1]. The etiology of ovarian cancer is multifactorial. Some studies suggest that the high grade serous types of ovarian cancer originates from the distal fallopian tube [2–4]. As a result, prophylactic bilateral salpingectomy during hysterectomy has been considered as a strategy to decrease the risk of high grade serous ovarian cancer [5, 6].

Some researchers have found that salpingectomy at the time of laparoscopic hysterectomy is a safe procedure for ovarian preservation [7–9]. Therefore, it is reasonable to consider that prophylactic bilateral salpingectomy may prevent ovarian cancer without the risk of premature menopause. However, other studies have found that bilateral salpingectomy alone or combined with hysterectomy could increase the risk of menopausal symptoms or decrease the antral follicle count (AFC) after surgery [10, 11]. Some researchers have expressed concern about postsurgical ovarian function since fallopian tubes and their surroundings might have contributed to the ovarian blood supply. The ovarian blood supply comes from the infundibulopelvic vessels, the ovarian branch of the uterine vessels, and the communicating branch formed by these. Laparoscopic hysterectomy and prophylactic salpingectomy can damage the the uterine branch and communicating branch close to fallopian tube [12]. It is not clear whether the infundibulopelvic vessels alone can guarantee ovarian reserve and whether prophylactic salpingectomy can affect ovarian function or menopause time. Patients are understandably concerned about the effects of bilateral salpingectomy, but relatively few studies have been conducted in China to assess the risks and benefits of the procedure.

Ovarian reserve may be influenced by age, genetics, and environmental factors [13–15]. Although there is no accepted direct measure of “ovarian reserve”, it is widely evaluated by multiple factors. Recently, markers such as sex hormones, serum anti-Müllerian hormone (AMH), and ultrasound AFC have been shown to provide a direct and accurate measurement of ovarian reserve [16–18]. Therefore, the purpose of this study was to assess the ovarian reserve of patients with prophylactic bilateral salpingectomy after laparoscopic hysterectomy by measuring serum AMH, follicle-stimulating hormone (FSH), estradiol (E2), and three-dimensional AFC. We also evaluated the differences in patient age, operative time, and estimated blood loss to determine if salpingectomy shows any additional risk beyond its potential benefits for patients as a novel strategy for decreasing the risk of ovarian cancer.

## Methods

This retrospective study investigated the relationship between prophylactic bilateral salpingectomy at the time of laparoscopic hysterectomy and ovarian function parameters in 373 Asian women by assessing serum AMH, FSH, E2, and AFC before and after surgery. The study was conducted at China Medical University, Shengjing Hospital, in the Department of Obstetrics and Gynecology, between January 2016 and March 2020. Records were reviewed for women aged 30 to 45 years with regular menstruation and laparoscopic hysterectomy with ovarian preservation for newly diagnosed and previously untreated benign uterine disease. Women with a personal or family history of malignant tumor, concurrent ovarian or tubal tumors or reproductive endocrinology-related diseases, failure to follow up after surgery, or incomplete medical records were excluded from the study. The upper age limit was chosen to prevent the selection of patients close to menopause. The clinical data for evaluating ovarian reserve, including serum AMH, FSH, E2, and three-dimensional AFC, were assessed before surgery and followed up twice at 3 and 9 months after surgery. Patients were divided according to whether they received laparoscopic hysterectomy alone (Group A, *n* = 202, ) or laparoscopic hysterectomy with prophylactic bilateral salpingectomy (Group B, *n* = 171). Laparoscopic hysterectomy with or without prophylactic bilateral salpingectomy was performed, generally the tubes were removed by ultrasound knife which coagulated and resected the mesosalpinx, beginning from the distal fimbrial extremity and proceeding toward the isthmus of fallopian tube.

Serum AMH, E2, and FSH were measured by the use of the commercially available chemiluminescence commercail kit (Beckman Coulter ) in the Medical Laboratory of Shengjing Hospital. AMH, FSH, E2, and AFC were measured in all patients during the first to fifth day of their menstrual cycle. The detection limit of AMH was 0.02-24 ng/mL. The intra- and inter-assay coefficients of variation (CVs) were below 5.5 and 4.5 %, respectively. The detection limit for FSH was 0.2–200 mIU/mL, and the intra- and inter-assay CVs were below 4.0 and 6 %, respectively. The detection limit of LH was 0.2–500 mIU/mL. The intra- and inter-assay CVs were below 5.5 and 4.5 %, respectively. The detection limit for E_2_ was 15.0-5200 pg/mL, with intra- and inter-assay CVs of 5 and 6 %, respectively. Patients were excluded from the group when their serum testing results were below the detectable limits before surgery. AFC was detected by the Department of Ultrasound in Shengjing Hospital. Secondary variables were obtained from medical records, including age, operative time, and estimated blood loss. The change in ovarian reserve after surgery was evaluated as the difference between postoperative and preoperative AMH, E2, FSH, LH, and AFC. The incidence of pelvic diseases in the two groups was assessed 3 and 9 months after surgery.

### Statistical analysis

Comparisons between continuous variables were analyzed using the Student’s t test or Wilcoxon rank-sum test. Statistical significance was defined as P < 0.05. Statistical analyses were performed using SPSS 13.1 (SPSS Inc., Chicago, USA).

## Results

As shown in Table 1, there was no significant difference at baseline between two groups in AMH, E2, FSH, LH, and AFC (all *p* > 0.05). As shown in Table 2, The two groups were also similar in age, operative time, and blood loss (all *p* > 0.05). Meanwhile, there was no significant difference between two groups 3 or 9 months after surgery in AMH, E2, FSH, LH, and AFC (all *p* > 0.05) (Tables 3 and 4).

**Table 1:** The basic factors of enrolled patients

	Non-salpingectomy group No.202	Salpingectomy group No. 171	*P* value
AGE	41.34 ± 2.67	41.54 ± 2.67	0.622
OPERATE TIME (minutes)	87.24 ± 40.56	92.89 ± 41.84	0.789
BLOOD LOSS (milliliters)	91.24 ± 47.43	86.90 ± 49.12	0.454

**Table 2:** Baseline assessments in Groups A and B

	Non-salpingectomy group A. No.202	Salpingectomy group B. No. 171	*P* value
Basal AMH (ng/ml)	0.85 ± 0.46	0.87 ± 0.47	0.379
Basal E2 (pg/ml)	88.32 ± 32.40	87.45 ± 30.99	0.645
Basal FSH (mIU/mL)	12.50 ± 4.58	12.04 ± 3.92	0.067
Basal LH (mIU/mL)	10.54 ± 2.23	10.33 ± 1.99	0.106
Antral follicle counts	6.47 ± 2.04	6.38 ± 2.53	0.550

**Table 3:** Month three and nine assessments in Groups A and B

	Non-salpingectomy group A No.202	Salpingectomy group B No. 171	*P* value
AMH (ng/ml)	-0.01 ± 0.43	0.00 ± 0.45	0.763
E2 (pg/ml)	1.79 ± 20.75	5.17 ± 34.60	0.264
FSH (mIU/mL)	−0.35 ± 3.55	−0.61 ± 3.61	0.478
LH (mIU/mL)	−0.34 ± 2.33	0.11 ± 2.44	0.07
Antral follicle counts	0.59 ± 3.48	−0.34 ± 2.33	0.061

**Table 4:** Nine months after surgery

	Salpingectomy group No. 171	Non-salpingectomy group No.202	*P* value
AMH (ng/ml)	-0.01 ± 0.55	-0.01 ± 0.49	0.939
E2 (pg/ml)	9.38 ± 34.81	4.70 ± 23.83	0.137
FSH (mIU/mL)	-0.65 ± 3.71	-1.08 ± 3.69	0.276
LH (mIU/mL)	0.17 ± 2.41	0.20 ± 2.43	0.070
Antral follicle counts	0.62 ± 4.10	-0.07 ± 2.89	0.066

At 9 months after surgery, no patients had malignant ovarian tumors. The incidences of benign ovarian tumors in the salpingectomy group 3 and 9 months after surgery were 0 % (0/373) and 2.68 % (1/373), respectively. The corresponding values in the hysterectomy alone group were 0 % (0/373) and 5.36 % (2/373), respectively. The incidences of pelvic inflammatory disease in the salpingectomy group 3 and 9 months after surgery were 10.72 % (4/373) and 8.04 % (3/373), respectively, while corresponding values in the hysterectomy alone group were 24.13 % (9/373) and 16.09 % (6/373), respectively.

## Discussion

Previous data suggested that high grade of serous ovarian carcinoma cells originate from the fallopian tube. Considering this theory, prophylactic salpingectomy has been proposed as a good method for preventing serous ovarian cancer since 2006 [19]. Recent clinical data have demonstrated that salpingectomy reduces the ovarian cancer risk better than other methods [20]. This is strongly suggested in further studies [8, 9, 21]. Ovarian reserve refers to the quantity and quality of follicles with normal developmental potential stored in the ovaries and reflects female fertility. As for Women of childbearing age, whether prophylactic salpingectomy during laparoscopic hysterectomy could damage ovarian reserve is uncertain.

This study was designed to evaluate the effect of prophylactic salpingectomy on ovarian reserve. It is an increasing concern as more women delay conception and can be predicted by multiple factors, including basic sex hormones, AMH, and the number of antral follicles. In this study, results showed that there were no significant postsurgical differences between the hysterectomy and hysterectomy with salpingectomy groups in serum AMH, FSH, E2, and three-dimensional AFC. Further, patients in these two groups were similar in terms of age, operation time, and blood loss. These results demonstrate that salpingectomy during laparoscopic hysterectomy did not damage the ovarian reserve 3 months and 9 months after surgery. Although the excision of the mesosalpinx ligament close to fallopian tube may damage the communicating branch of ovarian blood supply, our results suggest that other branches provide enough blood supply for ovary.

One limitation for our results is that we only followed up at three-month and nine-month points to detect the sexual hormone level after surgery. Many studies demonstrated that the third postoperative month is sufficient to assess the effect of surgery on acute ovarian reserve [7, 22, 23]. Further, the ninth-month secondary evaluation reinforces the accuracy of our results. However longer follow-up should be conducted. Since it could contribute much to the further effect on ovarian reserve, like whether there is anydifferent effect of age or environmental factors on ovarian reserve between two groups. The other possible limitation is that the mean age of women enrolled in this study was 41 years. It would be optimal to assess the effect of surgery on younger women, in whom ovaries have greater potential for follicle loss. However, younger patients are more likely to be concerned with maintaining reproductive ability and are less likely to undergo hysterectomy [24–26]. Further, delayed childbearing has increased in China in recent years and is very common globally [27, 28]. Therefore, the average age of patients in studies conducted to date on prophylactic salpingectomy during laparoscopic hysterectomy is > 37 years [29, 30].

We also followed the incidence of pelvic diseases 3 and 9 months after surgery. At 9 months after surgery, no patients had malignant ovarian tumors. The incidence of benign ovarian tumors in the two groups was low. The incidence of pelvic inflammatory disease in the salpingectomy group was lower than that in the hysterectomy alone group. Further study is required to determine whether salpingectomy will offer patients protection against carcinogenesis and prevention of ovarian cancers.

## Conclusions

Prophylactic bilateral salpingectomy does not damage the ovarian function of reproductive-age women who undergo laparoscopic hysterectomy. Moreover, salpingectomy is considered as a fesible and potentially effective risk-preventing choice, although further studies are needed to verify the benefits.

## Data Availability

The datasets generated and analyzed during the current study are not publicly available due patient privacy concerns but are available from the corresponding author on reasonable request.

## Abbreviations

AMH: Anti-Müllerian hormone
FSH: Follicle-stimulating hormone
LH: Luteinizing hormone
E2: Estradiol (E2)
AFC: Antral follicle count
CVs: Coefficients of variation
